# A Rare Case of Tracheal Lipoma in Puerto Rico: Presentation and Management of a Patient With Longstanding Respiratory Complaints

**DOI:** 10.7759/cureus.68010

**Published:** 2024-08-28

**Authors:** Giovanny E Perez Ortiz, Edgar F Del Toro Diez, Clariliz Munet Colon, Camila S Ríos de Choudens, Andres Gorbea, Shayanne Lajud

**Affiliations:** 1 Department of Otolaryngology - Head and Neck Surgery, University of Puerto Rico, Medical Sciences Campus, San Juan, PRI

**Keywords:** tracheal lipoma, airway disorders, multi-detector computed tomography (mdct), awake tracheostomy, lipoma, trachea

## Abstract

Tracheal lipomas, though exceedingly rare among benign tracheal masses, present unique diagnostic and management challenges due to their unusual clinical course. This case report documents the presentation and surgical management of a 56-year-old male with a tracheal lipoma, a first documented case in Puerto Rico. The patient presented with progressive dyspnea and stridor, initially misdiagnosed and treated as asthma exacerbations. Imaging confirmed a pedunculated tracheal mass, prompting emergency surgical intervention to secure the airway and excise the mass successfully. Histopathological analysis confirmed the mass as a benign tracheal lipoma. This case emphasizes the importance of considering rare tracheal tumors in the differential diagnosis of unexplained respiratory distress and advocates for tailored management strategies informed by comprehensive multidisciplinary approaches.

## Introduction

Respiratory distress refers to abnormal breathing patterns that cause patient discomfort, characterized by inspiratory or expiratory effort. It has many potential causes that must be urgently and thoroughly evaluated. Tracheal lipomas are uncommon airway masses and, as a result, are not usually the first diagnosis considered. In view that the majority of adult tracheal masses that cause airway obstruction are malignant in nature (80-90%), endotracheobronchial lipomas are extremely rare primary tracheal tumors that account for approximately 0.1% of benign lung masses [1,2]. Politis et al. noted in their seminal study that of 50 endotracheobronchial lipomas examined, only three were located within the trachea [3]. To our knowledge, the incidence of purely tracheal lipomas remains unknown. These benign masses usually have a pedunculated appearance that is believed to arise from the submucosal fat of the tracheobronchial tree between the cartilaginous rings. This is consistent with the histologic findings of mature adipocytes [4].

There are various established parameters regarding the treatment of obstructive airway masses. Securing the airway (e.g., tracheostomy) and performing interventional bronchoscopy are viable options for patients experiencing obstructive lesions of the respiratory tract, aimed at re-establishing the airway and alleviating symptoms [5]. This case report details the presentation and management of a patient with a tracheal lipoma, which resulted in respiratory distress. This is the first documented case of its kind in Puerto Rico, including details of our management strategy.

## Case presentation

A 56-year-old male arrived at our otolaryngology clinic with acute shortness of breath and audible stridor that had been present for the past six months. He also reported periodic episodes of dyspnea that originally were upon exertion but had progressed to occur while at rest. During this time frame, the patient's symptoms gradually worsened, leading to multiple visits to the emergency room where he was treated for asthma exacerbations and later discharged once symptoms were controlled. He denied chest pain, hemoptysis, weight changes, night sweats, chills, voice changes, dysphagia, or odynophagia. Our patient referred to have other comorbidities such as type 2 diabetes mellitus, hypertension, asthma, and gout. He did not refer to any toxic habits, such as smoking, or any abnormal social or occupational exposures.

Upon physical examination, the patient was an obese male who was hypertensive, tachypneic, and tachycardic with audible stridor, clear lung bases, and oxygen saturation at 92% with a nasal cannula (3 L/min). To evaluate the airway, 4% topical lidocaine was infiltrated transtracheal at the level of the cricothyroid membrane to numb the vocal cords. Fiberoptic visualization was performed, which revealed normal anatomy of the nasopharynx, oropharynx, hypopharynx, supraglottis, and glottis. Both true vocal cords were mobile. The scope was advanced into the subglottis where at approximately 4 cm from the true vocal cords, a round mucosal pedunculated mass was visualized arising from the left lateral tracheal wall with almost complete obstruction of the airway (Video 1).

**Video 1:** Flexible bronchoscopy with a view of a tracheal mass approximately 4 cm from the glottis.

**Figure 1:**
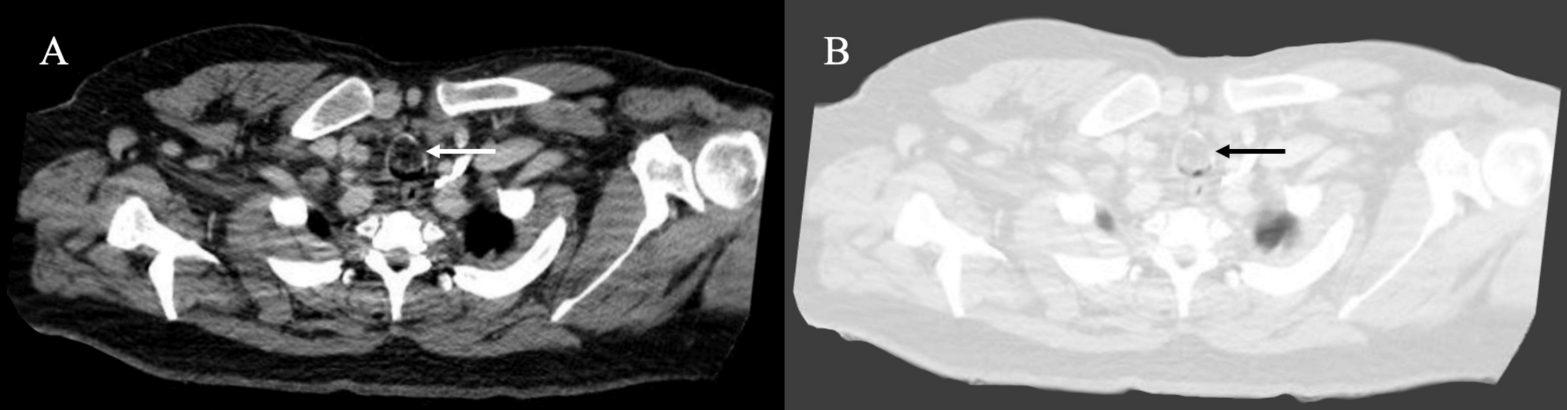
(A, B) Axial view of the CT revealing tracheal tumor in the abdomen and lung window, respectively (white and black arrows).

**Figure 2:**
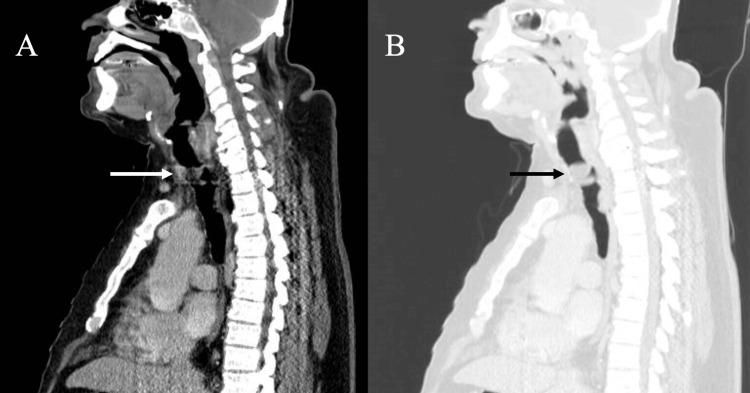
(A, B) Sagittal view of the CT revealing tracheal tumor in the abdomen and lung window, respectively (white and black arrows).

The patient underwent a multi-detector computed tomography (MDCT) scan of the neck and chest, which revealed a macroscopic fat containing lobulated mass, with enhancing peripheral nodularities located within the anterior aspect of the trachea 4 cm distal to the vocal cords at the level of C7 vertebrae. The mass measured approximately 2.2 x 2.2 x 1.7 cm (anterior-posterior × transverse × craniocaudal) with significant luminal narrowing (Figures 1, 2). No other significant lesions were visualized along the neck and chest.

**Figure 3:**
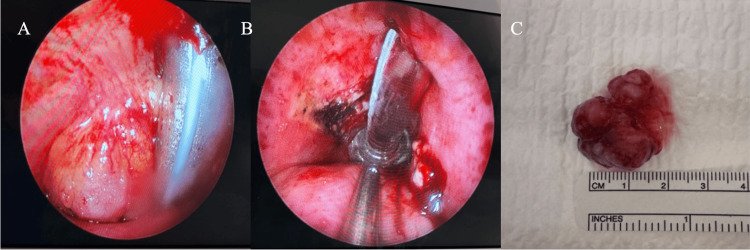
(A) Endoscopic view of the left tracheal wall prior to the resection of the mass. (B) Endoscopic view of the left tracheal wall post resection of the mass. (C) Lobulated exophytic tracheal mass.

Due to these findings, the patient was sent to the emergency department for immediate surgical management in the operating room. Our primary plan was to establish a secure airway. An awake tracheostomy with local anesthesia was performed and an endotracheal tube (ETT) was introduced through the trachea to bypass the mass and secure the airway. General anesthesia was later induced once placement was confirmed. Following this procedure, direct laryngoscopy with rigid bronchoscopy revealed a hard exophytic tracheal mass that was attached to the anterolateral aspect of the left side of the trachea approximately 4.0 cm from the glottis. Excision was performed in an en-bloc fashion with cup forceps, endoscopic scissors, and suction cautery. A frozen section was sent to the pathology and the tissue was reported negative for malignancy. This prompted our decision to undergo complete excision with special attention to the attachment site that was cauterized with suction cautery while the anesthesiologist decreased O2 supplementation to a fraction of inspired oxygen (FiO2) level < 30% (Figure 3). Once hemostasis was achieved, the fiberoptic lens was advanced through the airway up to the level of the carina, which revealed complete airway patency and no additional masses in the tracheal lumen. Finally, the ETT was exchanged for a cuffed tracheostomy tube (Covidien, Dublin, Ireland) and fixed in place.

Following surgical intervention, the patient was later admitted for observation. The patient had a relatively unremarkable postoperative course with no need for supplemental oxygen since postoperative day one. On postoperative day six, the final pathology report described the mass to be consistent with that of a tracheal lipoma. Following appropriate airway evaluation, the patient was decannulated on postoperative day eight and discharged home. The patient had an outpatient appointment approximately one month after discharge where he was asymptomatic. Flexible laryngoscopy and bronchoscopy performed elucidated expected wound healing with no signs of recurrence.

## Discussion

Tracheal lipomas have an indolent presentation with nonspecific progressive airway complaints, including coughing, wheezing, and/or shortness of breath [4]. These symptoms may lead to exertional dyspnea, orthopnea, and eventually dyspnea at rest. Our patient had a six-month history of fatigue and respiratory distress that affected his quality of life. The vagueness of these symptoms contributes to the patients presenting later in their disease timeline [6]. This is partly because the tracheal lumen has a large functional reserve, with tumors not causing symptoms until they occlude 50-75% of the lumen diameter [7]. Many patients with benign tracheal masses are often misdiagnosed and treated for chronic obstructive pulmonary diseases (asthma, emphysema, etc.), experiencing episodic improvement but frequent relapses.

Most tracheal lipomas, along with other benign tracheal masses, are typically diagnosed due to high clinical suspicion or incidentally during investigation for other suspected conditions. Our patient visited pulmonologists and internists who treated his symptoms as uncontrolled asthma. A viable workup option for our patient would have been pulmonary function testing. Fixed upper airway obstruction (e.g., tracheal mass) has a characteristic flattening of both inspiratory and expiratory phases on flow-volume loops [7]. This understanding might have led the pulmonologist to conduct imaging studies earlier, potentially preventing further airway compromise. Many of these patients do not respond completely to optimal medical management of common obstructive lung diseases [8]. Therefore, imaging studies are employed to further investigate potential causes and end up identifying these inconspicuous masses. Radiographic images, such as MDCT and MRI, are the most accurate noninvasive methods of airway evaluation, allowing diagnosis and treatment planning [5]. MDCT and MRI have the capability to recognize these tumors, assess their extent, and involvement with surrounding structures, and determine their composition. Fat can be detected with high specificity and sensitivity on MDCT, which in the setting of an endotracheobronchial mass can lead to the diagnosis of a tracheal lipoma [1]. The patient’s mass had no extra-tracheal involvement, circumscribed to a short segment of the trachea and with features suggestive of a lipoma.

In recent literature, different management strategies regarding tracheal lipomas have been described. Zhang et al. described their approach with a combination of endoscopic resection, tracheal stenting, and eventually tracheal resection with anastomosis [9]. Our approach resembles Basiari et al.'s strategy of stabilizing the airway with an awake tracheostomy prior to intervening on the mass [10]. The reasoning behind this stems from trying to perform the safest procedure that is most comfortable for the distressed patient and increases the chances of patient survival. In experienced hands, performing a tracheostomy and advancing an endotracheal tube to bypass the mass provides a rapid means of stabilizing and protecting the airway. Once the airway is secure, we believe the surgeon can continue the procedure in a calmer setting reducing the risks of complications.

Rigid bronchoscopy was then utilized to assess the mass for possible resection. At this point, malignancy is still high on our differential due to being the most common adult tracheal mass [2]. The general appearance of these tracheal lipomas usually resembles a polypoid lesion with a narrow attachment site [11]. These features are nonspecific and tissue biopsy is the gold standard to accurately reach a diagnosis in the majority of neoplasms. The frozen section reported the patient’s tissue being negative for malignancy. Taking all the patient’s factors into consideration (history, physical exam, imaging studies, and histology), we decided to remove the mass in its entirety.

Treatment options for endoluminal exophytic airway tumors generally depend on different factors such as the patient’s symptoms, the type of tumor, the degree of airway narrowing, and the local expertise available [12]. For this reason, numerous management options exist. Oberg et al. described the different options for managing malignant obstructive airway neoplasms by means of rigid/flexible bronchoscopic excision via laser, electrocautery, argon plasma coagulation, cryotherapy, mechanical debulking, microdebridation, and photodynamic therapy [5]. Recent literature describes managing the majority of obstructive endotracheobronchial lipomas with rigid bronchoscopy due to being a relatively safe procedure with a high success rate [8,9,13]. Our approach combined establishing a secure airway via a tracheostomy with bronchoscopic removal of the tumor. This method effectively secured the airway and allowed for successful tumor removal, demonstrating its efficacy as a management option. This strategy was selected due to our surgical background, which was instrumental in the procedure's success. Ultimately, documenting cases like these may aid in the development of management guidelines that can assist in the decision-making process for these complex patients.

## Conclusions

Tracheal lipomas and other benign tracheal tumors are very rare but possible causes of respiratory distress. This case emphasizes the importance of having tracheal tumors under consideration in patients with progressive dyspnea that does not respond to medical management. These cases require an extensive history, physical exam, imaging studies, and direct visualization. The rarity of this pathology and scarcity of guidelines render it significant to discuss the differences in management strategies to tailor care for future cases here in Puerto Rico and across the globe.
